# Editorial: The role of microglia in the pathogenesis of neurodegenerative diseases

**DOI:** 10.3389/fnagi.2022.1105896

**Published:** 2023-01-06

**Authors:** Qi Qin, Meng Wang, Huiliang Li, Zhiqing David Xu, Yi Tang

**Affiliations:** ^1^Department of Neurology and Innovation Center for Neurological Disorders, Xuanwu Hospital, Capital Medical University, Beijing, China; ^2^National Center for Neurological Disorders, Beijing, China; ^3^Wolfson Institute for Biomedical Research, University College London, London, United Kingdom; ^4^Department of Pathology, Capital Medical University, Beijing, China

**Keywords:** microglia, neurodegenerative diseases, disease-associated microglia, neuroinflammation, pathogenesis (nervous system)

Microglia, accounting for ~10–15% of glial cells in the brain, are the resident immune cells of the central nervous system (CNS), and play crucial roles in numerous biological processes, including immune surveillance, synapse pruning, and phagocytosis of dead cells, to maintain CNS stability (Lawson et al., 1990; Wu et al., 2014). When resting microglia are activated, released pro-inflammatory mediators and chemokines can regulate their activity and promote microglial phagocytosis of pathogens. Activated microglia also release anti-inflammatory factors, contributing to neuronal repair and anti-inflammatory defense (Schmid et al., 2009; Orihuela et al., 2016). However, upon adverse activation, microglia can produce neurotoxic substances, causing neuronal damage, accelerating the progression of neurodegenerative disease (ND).

Despite the lack of effective treatment for ND, relevant therapeutic options are being actively explored. To raise appreciation of microglia's role in ND, here we present to you three reviews and three original studies (three published in *Frontiers in Aging Neuroscience* and the other three in *Frontiers in Immunology*). These articles focus on the emerging roles of microglia in brain pathology, especially in ND, and provide inspiration for deciphering pathogenetic mechanisms of ND and developing microglia-targeted therapeutic strategies.

In a bibliometric study using VOSviewer, Sun et al. identify and analyze the top 100 most-cited original studies investigating the role of microglia in ND over the past 40 years. Alzheimer's disease (AD) proves to be the most studied ND in connection with microglial function, followed by Parkinson's disease (PD), and inflammation appears to be the current focal point. This study also reveals the most-cited authors, journals, institutions in this research field.

Microglia have proved able to regulate synaptic plasticity and synapse formation (Citri and Malenka, 2008; Hansen et al., 2018). It is widely believed that synaptic disruption contributes significantly to AD pathogenesis. In the review by Qin et al., the authors outline mechanisms for microglial synapse elimination, especially looking into the role of TREM2, which is a membrane receptor expressed in microglia and a recently identified genetic risk factor for AD. They propose that TREM2 interacts with APOE and C1q to trigger excessive microglial phagocytosis of synapses, contributing to synapse loss and impaired synaptic plasticity in AD.

TREM2 is exclusively expressed by microglia in the CNS (Zhou et al., 2020), and the extracellular soluble fragment of TREM2 (sTREM2) found in cerebrospinal fluid is considered a valuable biomarker for neurological diseases with underlying microglia-mediated neuroinflammation (Piccio et al., 2016). In the study by Mo et al., the levels of CSF sTREM2 are shown to be significantly higher in patients with PD than in healthy controls and have an inverse correlation with PD sleep scale scores in PD patients, suggesting that sTREM2 is a potential biomarker for PD diagnosis and progression. These data also hint at a role for microglia in PD onset and progression.

In addition to ND, microglia-mediated neuroinflammation features in other brain diseases. Sepsis associated encephalopathy is sepsis-related brain dysfunction that occurs without direct CNS infection or structural damage, manifesting as cognitive disorders (Opal, 2010; Bauer et al., 2016; Kikuchi et al., 2019). In the review by Yan et al., the authors describe the pathophysiological mechanisms of SAE and then zoom in on the role of microglia in SAE pathogenesis. Notably, they round off the review with an overview of new advances in research on targeting microglia for SAE treatment.

In recent years, an increasing number of epidemiological and experimental studies have revealed that various infections, such as with bacteria, viruses, fungi, and parasites, are a potential risk factor for ND (Alonso et al., 2015). Evidence linking infection to ND is reviewed by Tran et al. The authors explain how infectious agents can enter the brain via the nose-brain, lung-brain and gut-brain axes and how microglia and astrocytes are engaged in neuroinflammation in AD, and then present findings on pathogen-induced neuroinflammation in AD, PD and other neurological conditions, hinting that treating pathogen infection could have a therapeutic effect on ND by alleviating neuroinflammation.

Brain inflammation induced by hypoxic-ischemic insults plays an important part in the pathology of perinatal brain injury, with long-term consequences for brain functions (Ten et al., 2004; Hagberg et al., 2016). Pozo-Rodrigálvarez et al. reveal that postnatal hypoxic-ischemic injury to developing brain can cause long-lasting neurodegeneration and reactive gliosis in mouse hippocampus, persisting into adulthood. Using transgenic mouse models, they further show that signaling through receptor for complement peptide C3a, which is predominantly expressed by microglia after hypoxic-ischemic, can confer protection on neurons, reducing neurodegeneration and reactive gliosis. Furthermore, they experiment with intranasal administration of C3a for treatment of HI-induced brain injury, which provides a translational basis for finding new approaches to tackling brain inflammation and secondary neurodegeneration in neonatal brain injury.

In sum, this Research Topic highlights the importance of microglial activities to the pathogenesis and progression of ND. Microglia can act as our friend in health but foe in disease, and understanding all facets of this frenemy's actions will help us devise novel therapeutic interventions in the fight against ND.

## Author contributions

QQ and MW: conceptualization and writing—review and editing. HLL, ZX, and YT: conceptualization, writing—review and editing, supervision, and project administration. All authors read and approved the final manuscript.

## References

[B1] Alonso R. Pisa D. Marina A. I. Morato E. Rábano A. Rodal I.. (2015). Evidence for fungal infection in cerebrospinal fluid and brain tissue from patients with amyotrophic lateral sclerosis. Int. J. Biol. Sci. 11, 546–558. 10.7150/ijbs.11084 25892962 PMC4400386

[B2] Bauer M. Singer M. Opal S. M. Levy M. M. Deutschman C. S. Hotchkiss R. S.. (2016). The third international consensus definitions for sepsis and septic shock (sepsis-3). JAMA 315, 801–810. 10.1001/jama.2016.0287 26903338 PMC4968574

[B3] Citri A. Malenka R. C. (2008). Synaptic plasticity: multiple forms, functions, and mechanisms. Neuropsychopharmacology. 33, 18–41. 10.1038/sj.npp.1301559 17728696

[B4] Hagberg H. David Edwards A. Groenendaal F. (2016). Perinatal brain damage: the term infant. Neurobiol. Dis. 92(Pt A), 102–12. 10.1016/j.nbd.2015.09.011 26409031 PMC4915441

[B5] Hansen D. V. Hanson J. E. Sheng M. (2018). Microglia in Alzheimer's disease. J. Cell Biol. 217, 459–472. 10.1083/jcb.201709069 29196460 PMC5800817

[B6] Kikuchi D. S. Campos A. C. P. Qu H. Forrester S. J. Pagano R. L. Lassègue B.. (2019). Poldip2 mediates blood-brain barrier disruption in a model of sepsis-associated encephalopathy. J. Neuroinflammation. 16, 241. 10.1186/s12974-019-1575-4 31779628 PMC6883676

[B7] Lawson L. J. Perry V. H. Dri P. Gordon S. (1990). Heterogeneity in the distribution and morphology of microglia in the normal adult mouse brain. Neuroscience 39, 151–170. 10.1016/0306-4522(90)90229-W 2089275

[B8] Opal S. M. (2010). Endotoxins and other sepsis triggers. Contrib. Nephrol. 167, 14–24. 10.1159/000315915 20519895

[B9] Orihuela R. McPherson C. A. Harry G. J. (2016). Microglial M1/M2 polarization and metabolic states. Br. J. Pharmacol. 173, 649–665. 10.1111/bph.13139 25800044 PMC4742299

[B10] Piccio L. Deming Y. Del-Águila J. L. Ghezzi L. Holtzman D. M. Fagan A. M.. (2016). Cerebrospinal fluid soluble TREM2 is higher in Alzheimer disease and associated with mutation status. Acta Neuropathol. 131, 925–933. 10.1007/s00401-016-1533-5 26754641 PMC4867123

[B11] Schmid C. D. Melchior B. Masek K. Puntambekar S. S. Danielson P. E. Lo D. D. Differential gene expression in LPS/IFNγ activated microglia macrophages: in vitro versus in vivo. *J. Neurochem*. (2009). 109 Suppl 1(Suppl. 1), 117–125. 10.1111/j.1471-4159.2009.05984.x 19393017 PMC2766614

[B12] Ten V. S. Wu E. X. Tang H. Bradley-Moore M. Fedarau M. V. Ratner V. I.. (2004). Late measures of brain injury after neonatal hypoxia-ischemia in mice. Stroke 35, 2183–2188. 10.1161/01.STR.0000137768.25203.df 15272130

[B13] Wu H. M. Zhang L. F. Ding P. S. Liu Y. J. Wu X. Zhou J. N.. (2014). Microglial activation mediates host neuronal survival induced by neural stem cells. J Cell. Mol. Med. 18, 1300–1312. 10.1111/jcmm.12281 24725889 PMC4124015

[B14] Zhou Y. Song W. M. Andhey P. S. Swain A. Levy T. Miller K. R.. (2020). Human and mouse singlenucleus transcriptomics reveal TREM2-dependent and TREM2-independent cellular responses in Alzheimer's disease. Nat. Med. 26, 131–142. 10.1038/s41591-019-0695-9 31932797 PMC6980793

